# Identification of frailty-associated genes by coordination analysis of gene expression

**DOI:** 10.18632/aging.102875

**Published:** 2020-02-29

**Authors:** Youwen Zhang, Ioulia Chatzistamou, Hippokratis Kiaris

**Affiliations:** 1 Department of Drug Discovery and Biomedical Sciences, College of Pharmacy, University of South Carolina, Columbia, SC 29208, USA; 2 Department of Pathology, Microbiology and Immunology, School of Medicine, University of South Carolina, Columbia, SC 29209, USA; 3 Peromyscus Genetic Stock Center, University of South Carolina, Columbia, SC 29208, USA

**Keywords:** frailty, coordination analysis, transcriptome

## Abstract

Differential expression analyses provide powerful tools for the identification of genes playing a role in disease pathogenesis. Yet, such approaches are usually restricted by the high variation in expression profiles when primary specimens are analyzed. It is conceivable that with the assessment of the degree of coordination in gene expression as opposed to the magnitude of differential expression, we may obtain hints underscoring different biological and pathological states. Here we have analyzed a publicly available dataset related to frailty, a syndrome characterized by reduced responsiveness to stressors and exhibiting increased prevalence in the elderly. We evaluated the transcriptome that loses its coordination between the frailty and control groups and assessed the biological functions that are acquired in the former group. Among the top genes exhibiting the lowest correlation, at the whole transcriptome level, between the control and frailty groups were TSIX, BEST1 and ADAMTSL4. Processes related to immune response and regulation of cellular metabolism and the metabolism of macromolecules emerged in the frailty group. The proposed strategy confirms and extends earlier findings regarding the pathogenesis of frailty and provides a paradigm on how the diversity in expression profiles of primary specimens could be leveraged for target discovery.

## INTRODUCTION

Frailty is a clinical syndrome that is characterized by reduced responsiveness to stressors due to physiological decline in multiple organs and is associated with poor health outcomes including falls, incident disability, hospitalization, and mortality [1–4]. Frailty is usually studied in the elderly, yet it affects younger individuals as well, 45 – 64 years old [5, 6]. With the number of Americans aged 65 and older projected to double by 2060 [7] frailty consists of a condition with important implications in the quality of life of older individuals and overall healthcare management.

Despite that this condition is being recognized as a distinct clinical entity, our understanding of its pathogenetic mechanism remains limited. Comprehensive molecular studies at the whole transcriptome level, were only recently initiated underscoring the role of a proinflammatory response in the development of this condition [8, 9]. Despite this progress, additional research is imperative, both at the level of generation of new primary experimental data and at the level of application of novel analytical approaches, facilitating extraction of biologically relevant and clinically meaningful information.

Conventionally, gene expression analyses aim to identify differentially expressed genes in predefined experimental groups. In such analyses, the magnitude of over- or under-expression is considered indicative for the impact of the corresponding genes in the pathology of interest. Such strategies are frequently limited by the variation in expression between specimens which is particularly relevant when genetically diverse specimens are analyzed [10, 11]. To overcome these limitations, we have applied an alternative strategy in which samples were evaluated by comparing the correlation of expression of specific genes with the whole transcriptome, in different experimental groups [12, 13]. Coupling such analysis with publicly available gene ontology platforms [14–17] could identify changes in the transcriptome that would not be appreciated by conventional differential expression analysis. Furthermore, it could provide hints regarding the biological implications of such changes. For example, by focusing on the unfolded protein response (UPR) we were able to unveil specific functions of UPR branches and how they change during pathology [12, 13].

To apply this strategy to frailty we have reanalyzed publicly available data extracted from a comprehensive study that was performed in individuals that developed this syndrome [9].

## RESULTS AND DISCUSSION

By arbitrarily selecting at least 70 reads as the cut-off in the NOR group we identified 178 highly expressed transcripts. This limit was set for the convenience of the calculations and in theory could be increased indefinitely, provided that appropriate tools for computational analysis are developed. For the same reason specimens were assigned to only 2 groups, the NOR and the FRA groups, however additional sub-groups could be utilized, if a higher number of samples were available.

**Figure 1:**
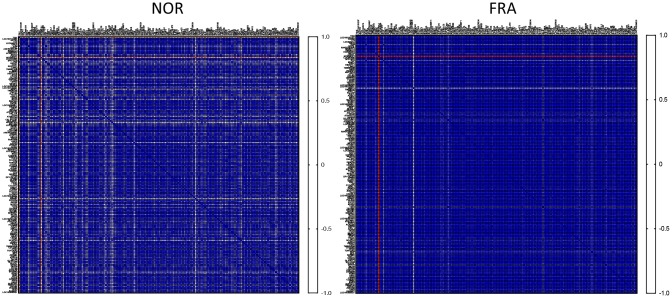
**Heatmaps of the correlation coefficients (R) among all pairwise comparisons between the most highly expressed genes in the NOR group.**

Initially, we asked how the expression among these 178 highly expressed genes is correlated between the NOR and FRA groups. To that end we calculated the correlation coefficient R (Pearson’s) for all pairwise comparisons between these 178 highly expressed genes, generating a heatmap illustrating the correlation in their expression. As shown in Figure 1, the vast majority of the genes subjected to this type of analysis was highly correlated with each other and the correlation increased in the FRA group. It is generally accepted that correlated expression or co-expression implies coregulation, by the same or similar transcription factors that define transcriptional networks [18–20]. According to the results of Figure 1, this coregulation becomes more intense during frailty. It is plausible that the lower degree of correlation in the control group (NOR) is indicative of the margins of expression at which physiological function for these genes can be attained. This flexibility is abolished in frailty because activation of signaling pathways under these conditions dictates more robust expression profiles. In line with this notion we recently reported that correlation was more intense in primary fibroblasts of outbred rodents, under endoplasmic reticulum stress as compared to unstressed cells in culture [21].

**Figure 2:**
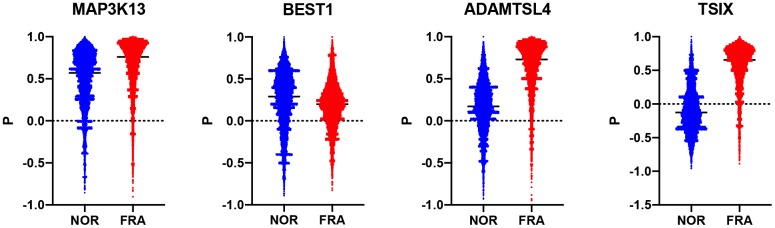
**Violin plots showing the R values between each of TSIX, BEST1, ADAMTSL4 and MAP3K13 in the NOR and the FRA groups.**

Subsequently, we estimated how the whole transcriptome is correlated with these 178 genes and compared how this correlation changes during frailty. To that end, a composite correlation (Pc) was calculated for each gene which corresponds to the correlation of the R values this gene has, with the whole transcriptome between the NOR and FRA groups. Then, we ranked these genes according to Pc (Supplementary Table 1). Therefore, high Pc indicates retention of coordination between the NOR and FRA groups while low Pc is suggestive for the loss of coordination, when the pathology emerges. The top 3 genes with lowest Pc were TSIX, BEST1 and ADAMTSL4 (-0.069, 0.074 and 0.135 respectively) while the top 3 with highest Pc were PNPT1, ORAI2 and MAP3K13 (0.462, 0.462 and 0.466 respectively) (Figure 2). These genes, such as TSIX, BEST1 and ADAMTSL4, are the ones that according to our hypotheses are being affected by (or affecting) frailty, or being affected minimally by this syndrome, such as PNPT1, ORAI2 and MAP3K13. TSIX encodes for an antisense RNA that is involved in the regulation of XIST and therefore in X chromosome inactivation [22, 23]. BEST1 encodes for a member of the bestrophin family of proteins that are calcium-activated chloride channels and have been associated with retinal disease [24, 25]. ADAMTSL4 participates in the formation of microfibrils and is associated with the development of *ectopia lentis*, an eye disorder [26].

**Figure 3:**
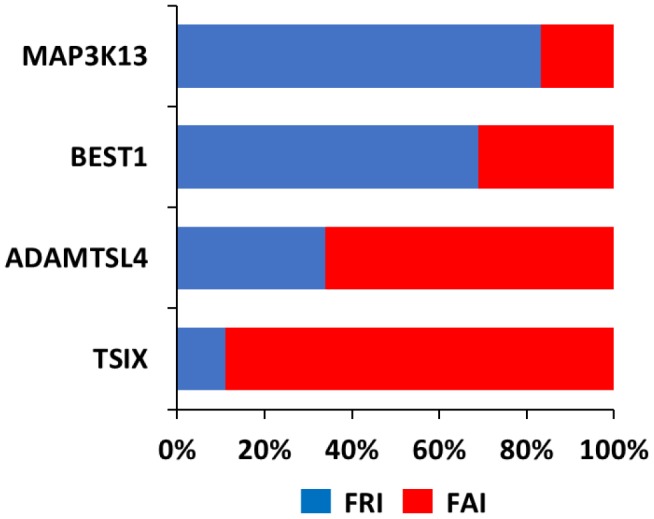
**Function Retention Index (FRI) and Function Acquisition Index (FAI) for each of TSIX, BEST1, ADAMTSL4 and MAP3K13.** FRI reflects the ratio of the functions in the NOR group that were retained in the FRA group (FRI=common functions in both groups/all functions in FRA group). FAI reflects the ratio of the novel functions in the FRA group that were absent from the NOR group (FAI=new functions in FRA group/all functions in FRA group).

In order to better understand the relevance of loss of coordination in TSIX, BEST1 and ADAMTSL4 we ranked the transcriptome according to its coordination with these 3 genes, Then, by using R=0.5 as a cut-off, we subjected the corresponding transcriptome to GO analysis [14, 15]. This analysis indicated that for the same gene, several functions were retained between the NOR and FRA groups, but several novel functions were also acquired (Figure 3 and Supplementary Table 2). Among the latter, the most prominent ones included functions related to immune system processes and metabolic processes (Table 1).

**Table 1:** Biological processes according to GO that were common for TSIX, BEST1 and ADAMTSL4 in the FRA group.

ADAMTSL4 in FRA, BEST1 in FRA, TSIX in FRA	103	immune system process (GO:0002376) regulation of cellular macromolecule biosynthetic process (GO:2000112) organic cyclic compound biosynthetic process (GO:1901362) cellular macromolecule metabolic process (GO:0044260) negative regulation of biosynthetic process (GO:0009890) cellular biosynthetic process (GO:0044249) negative regulation of RNA metabolic process (GO:0051253) cellular process (GO:0009987) macromolecule metabolic process (GO:0043170) positive regulation of macromolecule metabolic process (GO:0010604) negative regulation of RNA biosynthetic process (GO:1902679) negative regulation of transcription, DNA-templated (GO:0045892) detection of chemical stimulus involved in sensory perception of smell (GO:0050911) cellular component organization or biogenesis (GO:0071840) response to organic substance (GO:0010033) intracellular signal transduction (GO:0035556) detection of chemical stimulus involved in sensory perception (GO:0050907) nitrogen compound metabolic process (GO:0006807) regulation of RNA biosynthetic process (GO:2001141) positive regulation of cellular process (GO:0048522) cellular response to stress (GO:0033554) nucleic acid-templated transcription (GO:0097659) positive regulation of cellular metabolic process (GO:0031325) biological regulation (GO:0065007) positive regulation of transcription, DNA-templated (GO:0045893) positive regulation of nucleic acid-templated transcription (GO:1903508) RNA metabolic process (GO:0016070) biological process (GO:0008150) regulation of multicellular organismal process (GO:0051239) cellular aromatic compound metabolic process (GO:0006725) regulation of cellular process (GO:0050794) Unclassified (UNCLASSIFIED) organic cyclic compound metabolic process (GO:1901360) gene expression (GO:0010467) positive regulation of cellular biosynthetic process (GO:0031328) detection of chemical stimulus (GO:0009593) negative regulation of cellular macromolecule biosynthetic process (GO:2000113) cellular macromolecule biosynthetic process (GO:0034645) primary metabolic process (GO:0044238) biosynthetic process (GO:0009058) organic substance biosynthetic process (GO:1901576) cellular nitrogen compound metabolic process (GO:0034641) regulation of developmental process (GO:0050793) positive regulation of gene expression (GO:0010628) cellular response to stimulus (GO:0051716) regulation of macromolecule metabolic process (GO:0060255) positive regulation of protein metabolic process (GO:0051247) negative regulation of gene expression (GO:0010629) regulation of cellular metabolic process (GO:0031323) positive regulation of biological process (GO:0048518) positive regulation of nitrogen compound metabolic process (GO:0051173) metabolic process (GO:0008152) regulation of nitrogen compound metabolic process (GO:0051171) positive regulation of RNA biosynthetic process (GO:1902680) negative regulation of cellular process (GO:0048523) negative regulation of biological process (GO:0048519) apoptotic process (GO:0006915) negative regulation of macromolecule biosynthetic process (GO:0010558) macromolecule biosynthetic process (GO:0009059) localization (GO:0051179) regulation of transcription, DNA-templated (GO:0006355) nucleobase-containing compound metabolic process (GO:0006139) sensory perception of chemical stimulus (GO:0007606) hematopoietic or lymphoid organ development (GO:0048534) regulation of catalytic activity (GO:0050790) regulation of response to stress (GO:0080134) small molecule metabolic process (GO:0044281) negative regulation of nucleic acid-templated transcription (GO:1903507) detection of stimulus involved in sensory perception (GO:0050906) RNA biosynthetic process (GO:0032774) sensory perception of smell (GO:0007608) cellular component assembly (GO:0022607) positive regulation of macromolecule biosynthetic process (GO:0010557) cellular component organization (GO:0016043) regulation of cellular biosynthetic process (GO:0031326) immune system development (GO:0002520) positive regulation of metabolic process (GO:0009893) detection of stimulus (GO:0051606) regulation of metabolic process (GO:0019222) developmental process (GO:0032502) positive regulation of molecular function (GO:0044093) nucleic acid metabolic process (GO:0090304) leukocyte activation (GO:0045321) heterocycle biosynthetic process (GO:0018130) transcription, DNA- templated (GO:0006351) regulation of macromolecule biosynthetic process (GO:0010556) regulation of biological process (GO:0050789) regulation of gene expression (GO:0010468) regulation of nucleic acid-templated transcription (GO:1903506) cell activation (GO:0001775) regulation of nucleobase-containing compound metabolic process (GO:0019219) regulation of RNA metabolic process (GO:0051252) positive regulation of RNA metabolic process (GO:0051254) regulation of biosynthetic process (GO:0009889) cellular metabolic process (GO:0044237) heterocycle metabolic process (GO:0046483) sensory perception (GO:0007600) regulation of immune system process (GO:0002682) positive regulation of biosynthetic process (GO:0009891) regulation of primary metabolic process (GO:0080090) organic substance metabolic process (GO:0071704) regulation of molecular function (GO:0065009) positive regulation of nucleobase-containing compound metabolic process (GO:0045935)

These findings confirm and extend previous findings on the role of immune system in the pathogenesis of frailty and also identify the significance of metabolic deregulation or reprogramming in the development of this syndrome. In addition, they provide novel gene targets that may play a role in the development of this condition. It is conceivable that refinement of the proposed strategy, by including larger datasets and deeper and more expanded roster of genes to initiate the coregulation assessment, will be applicable to various conditions and be leveraged - as opposed to be restricted - by the high variation, when genetically diverse specimens are analyzed.

## MATERIALS AND METHODS

Data used were retrieved from GEO (Accession number: GSE129534). Specimens’ characteristics are described in detail in the original study [9]. Participants of the study were from the *Healthy Aging in Neighborhoods of Diversity across the Life Span* (HANDLS) study of the National Institute on Aging Intramural Research Program (NIA IRP), National Institutes of Health. In our analysis we assigned the specimens in 2 groups, with (FRA) or without (NOR) frailty, consistently with the classification of the original study [9]. Each group consisted of 8 samples, each of which included 4 whites and 4 African Americans, both males (50%) and females (50%). All individuals were 45-49 years old (Mean ± sd = 48.09 ±1.21 and 47.85 ± 1.84 for the NOR and FRA groups respectively). RNA-seq was performed in peripheral blood mononuclear cells [9].

**Figure 4:**
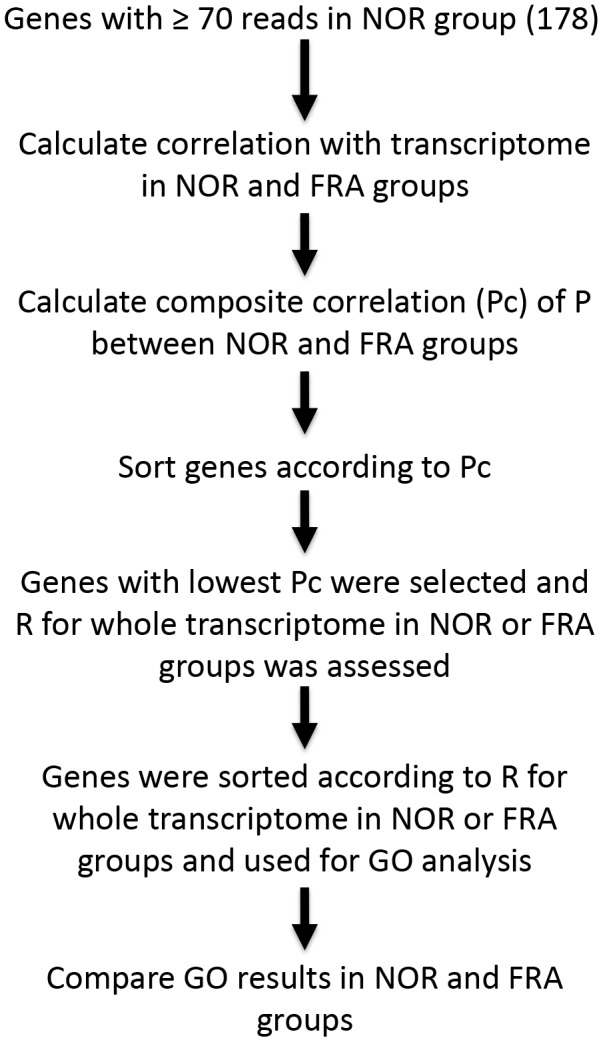
**Outline of the coordination analysis applied in the present study.**

The experimental outline we applied is shown in Figure 4. Initially we identified the transcripts exhibiting relatively high abundance. Arbitrarily we selected genes that displayed at least 70 reads in the NOR group (resulting in n=178 highly expressed genes). Subsequently we calculated the correlation (R, Pearson’s) for these 178 genes with the whole transcriptome, independently in the NOR and the FRA groups. In order to test for which of these genes correlation with the transcriptome changes in the different groups, we calculated the Pc from the R values calculated above. This transformation assigned a unique Pc value to each of these genes which reflects the degree by which coordination with the whole transcriptome changes in the 2 groups for the corresponding genes of interest. Then, the genes were sorted according to Pc, and for the ones that exhibited the lowest Pc (3 genes in this study) their correlation ® with the whole transcriptome, in the NOR and the FRA groups was calculated. These R values were used to sort the transcriptome and supply it to a GO platform for further analysis. As a cut-off we arbitrarily chose genes with R>0.5. Finally, predicted functions were compared between the NOR and the FRA groups for the genes selected.

## Supplementary Material

Supplementary Table 1

Supplementary Table 2
