# Supplementary material for: Identification of frailty-associated genes by coordination analysis of gene expression
Source: Aging (Albany NY). 2020 Feb 29;12(5):4222–9. doi: 10.18632/aging.102875 (PMC7093164; doi:10.18632/aging.102875)
Supplement: Supplementary Table 2 [file aging-12-102875-s001..docx]

**Supplementary Table 2.** Biological processes according to GO terms for each of TSIX, BEST1 and ADAMTSL4 in the NOR and FRA groups.

| **Gene** | **Number of Processes** | **Biological Processes** |
| --- | --- | --- |
| **TSIX** |  |  |
| TSIX FRA  TSIX NOR | 49 | cellular macromolecule metabolic process (GO:0044260) cellular protein metabolic process (GO:0044267) cellular process (GO:0009987) regulation of cell communication (GO:0010646) macromolecule metabolic process (GO:0043170) regulation of biological quality (GO:0065008) transport (GO:0006810) organonitrogen compound metabolic process (GO:1901564) cellular component organization or biogenesis (GO:0071840) detection of chemical stimulus involved in sensory perception (GO:0050907) nitrogen compound metabolic process (GO:0006807) positive regulation of cellular process (GO:0048522) biological regulation (GO:0065007) biological_process (GO:0008150) regulation of signaling (GO:0023051) regulation of protein phosphorylation (GO:0001932) macromolecule modification (GO:0043412) regulation of cellular process (GO:0050794) Unclassified (UNCLASSIFIED) regulation of cellular protein metabolic process (GO:0032268) primary metabolic process (GO:0044238) protein metabolic process (GO:0019538) regulation of macromolecule metabolic process (GO:0060255) regulation of cellular metabolic process (GO:0031323) positive regulation of biological process (GO:0048518) regulation of phosphorylation (GO:0042325) metabolic process (GO:0008152) regulation of nitrogen compound metabolic process (GO:0051171) regulation of cellular localization (GO:0060341) negative regulation of cellular process (GO:0048523) negative regulation of biological process (GO:0048519) regulation of protein metabolic process (GO:0051246) sensory perception of chemical stimulus (GO:0007606) detection of stimulus involved in sensory perception (GO:0050906) regulation of protein modification process (GO:0031399) regulation of intracellular signal transduction (GO:1902531) cellular response to chemical stimulus (GO:0070887) regulation of phosphate metabolic process (GO:0019220) positive regulation of metabolic process (GO:0009893) regulation of metabolic process (GO:0019222) regulation of biological process (GO:0050789) regulation of phosphorus metabolic process (GO:0051174) protein modification process (GO:0036211) negative regulation of cellular metabolic process (GO:0031324) cellular protein modification process (GO:0006464) cellular metabolic process (GO:0044237) regulation of primary metabolic process (GO:0080090) organic substance metabolic process (GO:0071704) regulation of molecular function (GO:0065009) |
| TSIX NOR | 1 | regulation of intracellular transport (GO:0032386) |
| TSIX FRA | 394 | complement activation (GO:0006956) positive regulation of intracellular signal transduction (GO:1902533) immune system process (GO:0002376) mRNA splicing, via spliceosome (GO:0000398) regulation of cellular macromolecule biosynthetic process (GO:2000112) epithelial cell differentiation (GO:0030855) organic cyclic compound biosynthetic process (GO:1901362) nuclear transport (GO:0051169) chromatin organization (GO:0006325) protein polyubiquitination (GO:0000209) positive regulation of apoptotic process (GO:0043065) negative regulation of biosynthetic process (GO:0009890) negative regulation of transcription by RNA polymerase II (GO:0000122) neutrophil activation (GO:0042119) mitotic cell cycle process (GO:1903047) cellular biosynthetic process (GO:0044249) regulation of Ras protein signal transduction (GO:0046578) G protein-coupled receptor signaling pathway (GO:0007186) macromolecule localization (GO:0033036) negative regulation of cellular catabolic process (GO:0031330) phosphorylation (GO:0016310) nucleobase-containing compound catabolic process (GO:0034655) regulation of autophagy (GO:0010506) cell cycle (GO:0007049) rRNA metabolic process (GO:0016072) negative regulation of translation (GO:0017148) establishment of localization in cell (GO:0051649) regulation of cell cycle (GO:0051726) amide biosynthetic process (GO:0043604) positive regulation of mitochondrion organization (GO:0010822) regulation of anatomical structure morphogenesis (GO:0022603) regulation of multicellular organismal development (GO:2000026) cellular response to DNA damage stimulus (GO:0006974) ubiquitin-dependent protein catabolic process (GO:0006511) viral gene expression (GO:0019080) keratinization (GO:0031424) negative regulation of RNA metabolic process (GO:0051253) regulation of catabolic process (GO:0009894) aromatic compound biosynthetic process (GO:0019438) regulation of signal transduction (GO:0009966) negative regulation of protein metabolic process (GO:0051248) B cell receptor signaling pathway (GO:0050853) cellular protein localization (GO:0034613) positive regulation of catabolic process (GO:0009896) negative regulation of signal transduction (GO:0009968) response to nitrogen compound (GO:1901698) regulation of cytokine production (GO:0001817) cell cycle process (GO:0022402) positive regulation of cell death (GO:0010942) positive regulation of macromolecule metabolic process (GO:0010604) nitrogen compound transport (GO:0071705) regulation of hemopoiesis (GO:1903706) nervous system process (GO:0050877) negative regulation of RNA biosynthetic process (GO:1902679) protein-containing complex assembly (GO:0065003) phosphate-containing compound metabolic process (GO:0006796) regulation of cell death (GO:0010941) positive regulation of signal transduction (GO:0009967) protein localization to endoplasmic reticulum (GO:0070972) organic substance transport (GO:0071702) histone modification (GO:0016570) nuclear export (GO:0051168) regulation of type I interferon production (GO:0032479) RNA splicing (GO:0008380) cellular nitrogen compound biosynthetic process (GO:0044271) protein-containing complex localization (GO:0031503) nuclear-transcribed mRNA catabolic process, nonsense-mediated decay (GO:0000184) negative regulation of transcription, DNA-templated (GO:0045892) positive regulation of response to external stimulus (GO:0032103) autophagy (GO:0006914) protein localization to organelle (GO:0033365) negative regulation of cell cycle process (GO:0010948) detection of chemical stimulus involved in sensory perception of smell (GO:0050911) regulation of apoptotic process (GO:0042981) translation (GO:0006412) response to organic substance (GO:0010033) nucleobase-containing compound transport (GO:0015931) carbohydrate derivative metabolic process (GO:1901135) intracellular signal transduction (GO:0035556) exocytosis (GO:0006887) translational initiation (GO:0006413) T cell activation (GO:0042110) protein modification by small protein conjugation (GO:0032446) process utilizing autophagic mechanism (GO:0061919) vesicle organization (GO:0016050) RNA localization (GO:0006403) regulation of cell cycle process (GO:0010564) regulation of RNA biosynthetic process (GO:2001141) protein phosphorylation (GO:0006468) cellular nitrogen compound catabolic process (GO:0044270) membrane invagination (GO:0010324) regulation of hydrolase activity (GO:0051336) regulation of cellular component size (GO:0032535) cellular response to stress (GO:0033554) mRNA metabolic process (GO:0016071) vesicle-mediated transport (GO:0016192) nucleic acid-templated transcription (GO:0097659) positive regulation of cellular metabolic process (GO:0031325) catabolic process (GO:0009056) interspecies interaction between organisms (GO:0044419) positive regulation of transcription, DNA-templated (GO:0045893) positive regulation of nucleic acid-templated transcription (GO:1903508) immune effector process (GO:0002252) regulation of RNA stability (GO:0043487) positive regulation of type I interferon production (GO:0032481) proteolysis involved in cellular protein catabolic process (GO:0051603) protein targeting to membrane (GO:0006612) regulation of cellular catabolic process (GO:0031329) leukocyte degranulation (GO:0043299) multi-organism process (GO:0051704) intracellular protein transport (GO:0006886) RNA metabolic process (GO:0016070) cellular macromolecule catabolic process (GO:0044265) keratinocyte differentiation (GO:0030216) regulation of complement activation (GO:0030449) regulation of multicellular organismal process (GO:0051239) regulation of programmed cell death (GO:0043067) cellular localization (GO:0051641) cellular aromatic compound metabolic process (GO:0006725) lymphocyte mediated immunity (GO:0002449) defense response to bacterium (GO:0042742) cellular response to organic substance (GO:0071310) regulation of protein kinase activity (GO:0045859) phagocytosis, engulfment (GO:0006911) organonitrogen compound catabolic process (GO:1901565) organic cyclic compound metabolic process (GO:1901360) establishment of organelle localization (GO:0051656) posttranscriptional regulation of gene expression (GO:0010608) protein localization to membrane (GO:0072657) organonitrogen compound biosynthetic process (GO:1901566) regulation of mRNA metabolic process (GO:1903311) gene expression (GO:0010467) peptide transport (GO:0015833) neutrophil mediated immunity (GO:0002446) neutrophil degranulation (GO:0043312) positive regulation of cellular biosynthetic process (GO:0031328) positive regulation of signaling (GO:0023056) humoral immune response mediated by circulating immunoglobulin (GO:0002455) ribonucleoprotein complex biogenesis (GO:0022613) detection of chemical stimulus (GO:0009593) regulation of apoptotic signaling pathway (GO:2001233) epidermal cell differentiation (GO:0009913) protein localization (GO:0008104) macroautophagy (GO:0016236) regulation of establishment of protein localization (GO:0070201) negative regulation of cellular macromolecule biosynthetic process (GO:2000113) aromatic compound catabolic process (GO:0019439) actin filament-based process (GO:0030029) plasma membrane invagination (GO:0099024) cellular macromolecule biosynthetic process (GO:0034645) negative regulation of signaling (GO:0023057) peptidyl-lysine modification (GO:0018205) cell recognition (GO:0008037) DNA repair (GO:0006281) biosynthetic process (GO:0009058) regulation of mitotic cell cycle (GO:0007346) regulation of multi-organism process (GO:0043900) organic substance biosynthetic process (GO:1901576) regulation of binding (GO:0051098) cellular nitrogen compound metabolic process (GO:0034641) establishment of protein localization to organelle (GO:0072594) modification-dependent macromolecule catabolic process (GO:0043632) regulation of developmental process (GO:0050793) regulation of mRNA processing (GO:0050684) positive regulation of gene expression (GO:0010628) organelle localization (GO:0051640) establishment of protein localization (GO:0045184) cellular response to stimulus (GO:0051716) regulated exocytosis (GO:0045055) cellular response to cytokine stimulus (GO:0071345) positive regulation of protein modification process (GO:0031401) cell activation involved in immune response (GO:0002263) RNA transport (GO:0050658) positive regulation of protein metabolic process (GO:0051247) negative regulation of gene expression (GO:0010629) negative regulation of cell communication (GO:0010648) RNA splicing, via transesterification reactions (GO:0000375) granulocyte activation (GO:0036230) positive regulation of response to stimulus (GO:0048584) positive regulation of multi-organism process (GO:0043902) lymphocyte differentiation (GO:0030098) immunoglobulin mediated immune response (GO:0016064) negative regulation of response to stimulus (GO:0048585) transcription by RNA polymerase II (GO:0006366) peptidyl-amino acid modification (GO:0018193) proteasomal protein catabolic process (GO:0010498) negative regulation of macromolecule metabolic process (GO:0010605) positive regulation of cellular component biogenesis (GO:0044089) establishment of RNA localization (GO:0051236) positive regulation of nitrogen compound metabolic process (GO:0051173) myeloid cell activation involved in immune response (GO:0002275) negative regulation of cellular biosynthetic process (GO:0031327) negative regulation of mitotic cell cycle (GO:0045930) vacuole organization (GO:0007033) RNA splicing, via transesterification reactions with bulged adenosine as nucleophile (GO:0000377) actin cytoskeleton organization (GO:0030036) humoral immune response (GO:0006959) proteolysis (GO:0006508) rRNA processing (GO:0006364) organelle organization (GO:0006996) peptide metabolic process (GO:0006518) viral transcription (GO:0019083) chromosome organization (GO:0051276) regulation of mRNA splicing, via spliceosome (GO:0048024) neutrophil activation involved in immune response (GO:0002283) epidermis development (GO:0008544) response to abiotic stimulus (GO:0009628) positive regulation of RNA biosynthetic process (GO:1902680) response to endoplasmic reticulum stress (GO:0034976) regulation of protein serine/threonine kinase activity (GO:0071900) SRP-dependent cotranslational protein targeting to membrane (GO:0006614) protein targeting to ER (GO:0045047) apoptotic process (GO:0006915) secretion (GO:0046903) cytokine-mediated signaling pathway (GO:0019221) regulation of mitotic cell cycle phase transition (GO:1901990) positive regulation of cellular protein metabolic process (GO:0032270) negative regulation of macromolecule biosynthetic process (GO:0010558) negative regulation of metabolic process (GO:0009892) mitotic cell cycle (GO:0000278) peptide biosynthetic process (GO:0043043) regulation of protein stability (GO:0031647) regulation of response to stimulus (GO:0048583) macromolecule biosynthetic process (GO:0009059) localization (GO:0051179) regulation of transcription, DNA-templated (GO:0006355) regulation of cellular amide metabolic process (GO:0034248) cellular amide metabolic process (GO:0043603) phosphorus metabolic process (GO:0006793) ncRNA processing (GO:0034470) regulation of cellular protein localization (GO:1903827) RNA processing (GO:0006396) nucleobase-containing compound metabolic process (GO:0006139) protein-containing complex disassembly (GO:0032984) ribosome biogenesis (GO:0042254) mRNA processing (GO:0006397) negative regulation of catabolic process (GO:0009895) regulation of cellular component biogenesis (GO:0044087) negative regulation of cell cycle (GO:0045786) B cell mediated immunity (GO:0019724) regulation of mRNA catabolic process (GO:0061013) regulation of organelle organization (GO:0033043) response to stress (GO:0006950) protein-containing complex subunit organization (GO:0043933) hematopoietic or lymphoid organ development (GO:0048534) regulation of catalytic activity (GO:0050790) post-Golgi vesicle-mediated transport (GO:0006892) regulation of response to stress (GO:0080134) small molecule metabolic process (GO:0044281) negative regulation of nucleic acid-templated transcription (GO:1903507) cell division (GO:0051301) ribonucleoprotein complex subunit organization (GO:0071826) negative regulation of organelle organization (GO:0010639) regulation of protein localization (GO:0032880) RNA biosynthetic process (GO:0032774) regulation of humoral immune response (GO:0002920) regulation of response to external stimulus (GO:0032101) cellular component biogenesis (GO:0044085) ncRNA metabolic process (GO:0034660) cotranslational protein targeting to membrane (GO:0006613) sensory perception of smell (GO:0007608) cellular catabolic process (GO:0044248) regulation of translation (GO:0006417) cellular component assembly (GO:0022607) regulation of cell cycle phase transition (GO:1901987) negative regulation of nitrogen compound metabolic process (GO:0051172) regulation of defense response (GO:0031347) regulation of chromosome organization (GO:0033044) positive regulation of macromolecule biosynthetic process (GO:0010557) cellular component organization (GO:0016043) macromolecule catabolic process (GO:0009057) regulation of cellular biosynthetic process (GO:0031326) regulation of signal transduction by p53 class mediator (GO:1901796) myeloid leukocyte activation (GO:0002274) modification-dependent protein catabolic process (GO:0019941) hemopoiesis (GO:0030097) organic substance catabolic process (GO:1901575) response to stimulus (GO:0050896) immune system development (GO:0002520) nucleobase-containing compound biosynthetic process (GO:0034654) positive regulation of cell communication (GO:0010647) cellular protein-containing complex assembly (GO:0034622) positive regulation of programmed cell death (GO:0043068) phagocytosis, recognition (GO:0006910) ribonucleoprotein complex assembly (GO:0022618) regulation of transferase activity (GO:0051338) protein catabolic process (GO:0030163) leukocyte activation involved in immune response (GO:0002366) viral process (GO:0016032) endomembrane system organization (GO:0010256) detection of stimulus (GO:0051606) regulation of cellular component organization (GO:0051128) positive regulation of protein kinase activity (GO:0045860) positive regulation of innate immune response (GO:0045089) regulation of cytoskeleton organization (GO:0051493) positive regulation of kinase activity (GO:0033674) positive regulation of hydrolase activity (GO:0051345) response to organonitrogen compound (GO:0010243) positive regulation of cellular component organization (GO:0051130) protein modification by small protein conjugation or removal (GO:0070647) DNA-dependent DNA replication (GO:0006261) cornification (GO:0070268) developmental process (GO:0032502) response to oxygen-containing compound (GO:1901700) positive regulation of molecular function (GO:0044093) system process (GO:0003008) positive regulation of GTPase activity (GO:0043547) positive regulation of cellular catabolic process (GO:0031331) heterocycle catabolic process (GO:0046700) regulation of protein complex assembly (GO:0043254) cytoskeleton organization (GO:0007010) establishment of protein localization to membrane (GO:0090150) nucleic acid metabolic process (GO:0090304) leukocyte activation (GO:0045321) heterocycle biosynthetic process (GO:0018130) DNA metabolic process (GO:0006259) regulation of mitochondrion organization (GO:0010821) proteasome-mediated ubiquitin-dependent protein catabolic process (GO:0043161) transcription, DNA-templated (GO:0006351) regulation of mRNA stability (GO:0043488) nuclear-transcribed mRNA catabolic process (GO:0000956) regulation of macromolecule biosynthetic process (GO:0010556) organelle assembly (GO:0070925) Golgi vesicle transport (GO:0048193) regulation of cellular response to stress (GO:0080135) nucleic acid transport (GO:0050657) nucleocytoplasmic transport (GO:0006913) regulation of GTPase activity (GO:0043087) symbiotic process (GO:0044403) establishment of protein localization to endoplasmic reticulum (GO:0072599) amide transport (GO:0042886) regulation of gene expression (GO:0010468) negative regulation of cellular protein metabolic process (GO:0032269) regulation of nucleic acid-templated transcription (GO:1903506) cell activation (GO:0001775) positive regulation of protein serine/threonine kinase activity (GO:0071902) regulation of nucleobase-containing compound metabolic process (GO:0019219) positive regulation of catalytic activity (GO:0043085) response to cytokine (GO:0034097) regulation of kinase activity (GO:0043549) negative regulation of intracellular signal transduction (GO:1902532) positive regulation of defense response (GO:0031349) negative regulation of cellular component organization (GO:0051129) negative regulation of cell death (GO:0060548) export from cell (GO:0140352) positive regulation of protein complex assembly (GO:0031334) intracellular transport (GO:0046907) regulation of localization (GO:0032879) regulation of RNA metabolic process (GO:0051252) cellular protein catabolic process (GO:0044257) regulation of small GTPase mediated signal transduction (GO:0051056) organic cyclic compound catabolic process (GO:1901361) homeostatic process (GO:0042592) positive regulation of RNA metabolic process (GO:0051254) regulation of biosynthetic process (GO:0009889) RNA catabolic process (GO:0006401) regulation of DNA metabolic process (GO:0051052) skin development (GO:0043588) regulation of RNA splicing (GO:0043484) positive regulation of autophagy (GO:0010508) cellular macromolecule localization (GO:0070727) regulation of gene expression, epigenetic (GO:0040029) DNA conformation change (GO:0071103) myeloid leukocyte mediated immunity (GO:0002444) covalent chromatin modification (GO:0016569) positive regulation of response to biotic stimulus (GO:0002833) establishment of localization (GO:0051234) protein targeting (GO:0006605) heterocycle metabolic process (GO:0046483) sensory perception (GO:0007600) adaptive immune response (GO:0002250) regulation of immune system process (GO:0002682) regulation of innate immune response (GO:0045088) lymphocyte activation (GO:0046649) positive regulation of biosynthetic process (GO:0009891) positive regulation of multicellular organismal process (GO:0051240) negative regulation of nucleobase-containing compound metabolic process (GO:0045934) negative regulation of cellular amide metabolic process (GO:0034249) positive regulation of transferase activity (GO:0051347) regulation of response to biotic stimulus (GO:0002831) protein transport (GO:0015031) organophosphate metabolic process (GO:0019637) regulation of I-kappaB kinase/NF-kappaB signaling (GO:0043122) protein ubiquitination (GO:0016567) DNA replication (GO:0006260) positive regulation of organelle organization (GO:0010638) secretion by cell (GO:0032940) complement activation, classical pathway (GO:0006958) positive regulation of cellular protein localization (GO:1903829) regulation of transcription by RNA polymerase II (GO:0006357) mRNA catabolic process (GO:0006402) chromatin remodeling (GO:0006338) regulation of macroautophagy (GO:0016241) positive regulation of nucleobase-containing compound metabolic process (GO:0045935) |
| **BEST1** |  |  |
| BEST1 FRA  BEST1 NOR | 78 | negative regulation of cellular process (GO:0048523) regulation of cellular macromolecule biosynthetic process (GO:2000112) cellular macromolecule metabolic process (GO:0044260) negative regulation of biological process (GO:0048519) cellular biosynthetic process (GO:0044249) macromolecule biosynthetic process (GO:0009059) localization (GO:0051179) regulation of transcription, DNA-templated (GO:0006355) nucleobase-containing compound metabolic process (GO:0006139) sensory perception of chemical stimulus (GO:0007606) cellular process (GO:0009987) regulation of catalytic activity (GO:0050790) macromolecule metabolic process (GO:0043170) regulation of response to stress (GO:0080134) small molecule metabolic process (GO:0044281) detection of stimulus involved in sensory perception (GO:0050906) positive regulation of macromolecule metabolic process (GO:0010604) sensory perception of smell (GO:0007608) cellular component assembly (GO:0022607) detection of chemical stimulus involved in sensory perception of smell (GO:0050911) cellular component organization or biogenesis (GO:0071840) positive regulation of macromolecule biosynthetic process (GO:0010557) cellular component organization (GO:0016043) response to organic substance (GO:0010033) regulation of cellular biosynthetic process (GO:0031326) intracellular signal transduction (GO:0035556) detection of chemical stimulus involved in sensory perception (GO:0050907) nitrogen compound metabolic process (GO:0006807) regulation of RNA biosynthetic process (GO:2001141) positive regulation of cellular process (GO:0048522) cellular response to stress (GO:0033554) positive regulation of metabolic process (GO:0009893) detection of stimulus (GO:0051606) positive regulation of cellular metabolic process (GO:0031325) regulation of metabolic process (GO:0019222) biological regulation (GO:0065007) positive regulation of molecular function (GO:0044093) nucleic acid metabolic process (GO:0090304) leukocyte activation (GO:0045321) RNA metabolic process (GO:0016070) biological_process (GO:0008150) regulation of macromolecule biosynthetic process (GO:0010556) regulation of biological process (GO:0050789) cellular aromatic compound metabolic process (GO:0006725) regulation of cellular process (GO:0050794) regulation of gene expression (GO:0010468) regulation of nucleic acid-templated transcription (GO:1903506) Unclassified (UNCLASSIFIED) cell activation (GO:0001775) organic cyclic compound metabolic process (GO:1901360) regulation of nucleobase-containing compound metabolic process (GO:0019219) gene expression (GO:0010467) positive regulation of cellular biosynthetic process (GO:0031328) regulation of RNA metabolic process (GO:0051252) detection of chemical stimulus (GO:0009593) positive regulation of RNA metabolic process (GO:0051254) regulation of biosynthetic process (GO:0009889) cellular metabolic process (GO:0044237) cellular macromolecule biosynthetic process (GO:0034645) primary metabolic process (GO:0044238) biosynthetic process (GO:0009058) organic substance biosynthetic process (GO:1901576) heterocycle metabolic process (GO:0046483) cellular nitrogen compound metabolic process (GO:0034641) sensory perception (GO:0007600) regulation of immune system process (GO:0002682) positive regulation of biosynthetic process (GO:0009891) regulation of macromolecule metabolic process (GO:0060255) regulation of primary metabolic process (GO:0080090) negative regulation of gene expression (GO:0010629) organic substance metabolic process (GO:0071704) regulation of cellular metabolic process (GO:0031323) positive regulation of biological process (GO:0048518) regulation of molecular function (GO:0065009) positive regulation of nitrogen compound metabolic process (GO:0051173) metabolic process (GO:0008152) regulation of nitrogen compound metabolic process (GO:0051171) positive regulation of nucleobase-containing compound metabolic process (GO:0045935) |
| BEST1 NOR | 116 | complement activation (GO:0006956) regulation of protein metabolic process (GO:0051246) cellular protein metabolic process (GO:0044267) regulation of Ras protein signal transduction (GO:0046578) G protein-coupled receptor signaling pathway (GO:0007186) macromolecule localization (GO:0033036) organic acid metabolic process (GO:0006082) negative regulation of metabolic process (GO:0009892) phosphorylation (GO:0016310) nucleobase-containing compound catabolic process (GO:0034655) regulation of response to stimulus (GO:0048583) cell cycle (GO:0007049) regulation of cellular amide metabolic process (GO:0034248) phosphorus metabolic process (GO:0006793) RNA processing (GO:0006396) regulation of mRNA catabolic process (GO:0061013) keratinization (GO:0031424) response to stress (GO:0006950) regulation of catabolic process (GO:0009894) regulation of signal transduction (GO:0009966) regulation of cell communication (GO:0010646) cellular protein localization (GO:0034613) nitrogen compound transport (GO:0071705) regulation of response to external stimulus (GO:0032101) nervous system process (GO:0050877) cellular component biogenesis (GO:0044085) phosphate-containing compound metabolic process (GO:0006796) regulation of cell death (GO:0010941) positive regulation of signal transduction (GO:0009967) organic substance transport (GO:0071702) cellular catabolic process (GO:0044248) regulation of biological quality (GO:0065008) cellular nitrogen compound biosynthetic process (GO:0044271) nuclear-transcribed mRNA catabolic process, nonsense-mediated decay (GO:0000184) activation of innate immune response (GO:0002218) positive regulation of response to external stimulus (GO:0032103) protein localization to organelle (GO:0033365) transport (GO:0006810) regulation of intracellular signal transduction (GO:1902531) organonitrogen compound metabolic process (GO:1901564) regulation of defense response (GO:0031347) regulation of apoptotic process (GO:0042981) macromolecule catabolic process (GO:0009057) organic substance catabolic process (GO:1901575) protein modification by small protein conjugation (GO:0032446) positive regulation of cell communication (GO:0010647) phagocytosis, recognition (GO:0006910) regulation of transferase activity (GO:0051338) protein phosphorylation (GO:0006468) cellular nitrogen compound catabolic process (GO:0044270) leukocyte activation involved in immune response (GO:0002366) mRNA metabolic process (GO:0016071) regulation of cellular component organization (GO:0051128) positive regulation of innate immune response (GO:0045089) catabolic process (GO:0009056) protein modification by small protein conjugation or removal (GO:0070647) innate immune response-activating signal transduction (GO:0002758) heterocycle catabolic process (GO:0046700) protein targeting to membrane (GO:0006612) regulation of cellular catabolic process (GO:0031329) establishment of protein localization to membrane (GO:0090150) intracellular protein transport (GO:0006886) cellular macromolecule catabolic process (GO:0044265) regulation of mRNA stability (GO:0043488) nuclear-transcribed mRNA catabolic process (GO:0000956) regulation of signaling (GO:0023051) keratinocyte differentiation (GO:0030216) macromolecule modification (GO:0043412) amide transport (GO:0042886) cellular localization (GO:0051641) regulation of programmed cell death (GO:0043067) posttranscriptional regulation of gene expression (GO:0010608) protein localization to membrane (GO:0072657) positive regulation of catalytic activity (GO:0043085) regulation of mRNA metabolic process (GO:1903311) organonitrogen compound biosynthetic process (GO:1901566) regulation of kinase activity (GO:0043549) response to cytokine (GO:0034097) peptide transport (GO:0015833) protein modification process (GO:0036211) positive regulation of defense response (GO:0031349) positive regulation of signaling (GO:0023056) intracellular transport (GO:0046907) humoral immune response mediated by circulating immunoglobulin (GO:0002455) regulation of localization (GO:0032879) regulation of cellular protein metabolic process (GO:0032268) regulation of small GTPase mediated signal transduction (GO:0051056) organic cyclic compound catabolic process (GO:1901361) RNA catabolic process (GO:0006401) epidermal cell differentiation (GO:0009913) protein localization (GO:0008104) cellular protein modification process (GO:0006464) cellular macromolecule localization (GO:0070727) aromatic compound catabolic process (GO:0019439) protein metabolic process (GO:0019538) positive regulation of response to biotic stimulus (GO:0002833) establishment of localization (GO:0051234) regulation of multi-organism process (GO:0043900) regulation of innate immune response (GO:0045088) establishment of protein localization (GO:0045184) organelle localization (GO:0051640) lymphocyte activation (GO:0046649) cell activation involved in immune response (GO:0002263) small molecule catabolic process (GO:0044282) positive regulation of response to stimulus (GO:0048584) positive regulation of multi-organism process (GO:0043902) regulation of response to biotic stimulus (GO:0002831) negative regulation of response to stimulus (GO:0048585) protein transport (GO:0015031) peptidyl-amino acid modification (GO:0018193) negative regulation of macromolecule metabolic process (GO:0010605) protein ubiquitination (GO:0016567) complement activation, classical pathway (GO:0006958) organelle organization (GO:0006996) mRNA catabolic process (GO:0006402) oxoacid metabolic process (GO:0043436) |
| BEST1 FRA | 35 | positive regulation of RNA biosynthetic process (GO:1902680) immune system process (GO:0002376) response to cadmium ion (GO:0046686) organic cyclic compound biosynthetic process (GO:1901362) negative regulation of biosynthetic process (GO:0009890) apoptotic process (GO:0006915) negative regulation of macromolecule biosynthetic process (GO:0010558) negative regulation of RNA metabolic process (GO:0051253) hematopoietic or lymphoid organ development (GO:0048534) negative regulation of nucleic acid-templated transcription (GO:1903507) RNA biosynthetic process (GO:0032774) positive regulation of NF-kappaB transcription factor activity (GO:0051092) negative regulation of RNA biosynthetic process (GO:1902679) multicellular organism development (GO:0007275) negative regulation of transcription, DNA-templated (GO:0045892) cell death (GO:0008219) negative regulation of immune system process (GO:0002683) in utero embryonic development (GO:0001701) immune system development (GO:0002520) nucleic acid-templated transcription (GO:0097659) positive regulation of transcription, DNA-templated (GO:0045893) positive regulation of nucleic acid-templated transcription (GO:1903508) developmental process (GO:0032502) heterocycle biosynthetic process (GO:0018130) transcription, DNA-templated (GO:0006351) regulation of DNA-binding transcription factor activity (GO:0051090) regulation of multicellular organismal process (GO:0051239) protein tetramerization (GO:0051262) protein heterotetramerization (GO:0051290) negative regulation of cellular macromolecule biosynthetic process (GO:2000113) protein heterooligomerization (GO:0051291) regulation of developmental process (GO:0050793) positive regulation of gene expression (GO:0010628) cellular response to stimulus (GO:0051716) positive regulation of protein metabolic process (GO:0051247) |
| **ADAMTSL4** |  |  |
| ADAMTSL4 FRA ADAMTSL4 NOR | 162 | positive regulation of intracellular signal transduction (GO:1902533) immune system process (GO:0002376) regulation of cellular macromolecule biosynthetic process (GO:2000112) cellular macromolecule metabolic process (GO:0044260) organic cyclic compound biosynthetic process (GO:1901362) positive regulation of apoptotic process (GO:0043065) neutrophil activation (GO:0042119) cellular biosynthetic process (GO:0044249) macromolecule localization (GO:0033036) cell cycle (GO:0007049) keratinization (GO:0031424) cellular process (GO:0009987) regulation of catabolic process (GO:0009894) aromatic compound biosynthetic process (GO:0019438) regulation of signal transduction (GO:0009966) regulation of cell communication (GO:0010646) macromolecule metabolic process (GO:0043170) negative regulation of protein metabolic process (GO:0051248) protein modification by small protein removal (GO:0070646) positive regulation of cell death (GO:0010942) regulation of cytokine production (GO:0001817) positive regulation of macromolecule metabolic process (GO:0010604) nitrogen compound transport (GO:0071705) positive regulation of cytokine production (GO:0001819) protein-containing complex assembly (GO:0065003) phosphate-containing compound metabolic process (GO:0006796) regulation of cell death (GO:0010941) positive regulation of signal transduction (GO:0009967) organic substance transport (GO:0071702) cellular response to oxygen-containing compound (GO:1901701) regulation of biological quality (GO:0065008) cellular nitrogen compound biosynthetic process (GO:0044271) positive regulation of response to external stimulus (GO:0032103) transport (GO:0006810) organonitrogen compound metabolic process (GO:1901564) detection of chemical stimulus involved in sensory perception of smell (GO:0050911) regulation of apoptotic process (GO:0042981) cellular component organization or biogenesis (GO:0071840) response to organic substance (GO:0010033) intracellular signal transduction (GO:0035556) detection of chemical stimulus involved in sensory perception (GO:0050907) nitrogen compound metabolic process (GO:0006807) vesicle organization (GO:0016050) regulation of RNA biosynthetic process (GO:2001141) positive regulation of cellular process (GO:0048522) cellular response to stress (GO:0033554) vesicle-mediated transport (GO:0016192) positive regulation of cellular metabolic process (GO:0031325) catabolic process (GO:0009056) biological regulation (GO:0065007) immune effector process (GO:0002252) regulation of immune response (GO:0050776) regulation of cellular catabolic process (GO:0031329) leukocyte degranulation (GO:0043299) cellular macromolecule catabolic process (GO:0044265) biological_process (GO:0008150) regulation of signaling (GO:0023051) cellular aromatic compound metabolic process (GO:0006725) regulation of programmed cell death (GO:0043067) cellular localization (GO:0051641) regulation of cellular process (GO:0050794) cellular response to organic substance (GO:0071310) Unclassified (UNCLASSIFIED) organic cyclic compound metabolic process (GO:1901360) neutrophil mediated immunity (GO:0002446) neutrophil degranulation (GO:0043312) regulation of protein transport (GO:0051223) positive regulation of signaling (GO:0023056) regulation of cellular protein metabolic process (GO:0032268) detection of chemical stimulus (GO:0009593) protein localization (GO:0008104) regulation of establishment of protein localization (GO:0070201) cellular macromolecule biosynthetic process (GO:0034645) primary metabolic process (GO:0044238) biosynthetic process (GO:0009058) regulation of multi-organism process (GO:0043900) organic substance biosynthetic process (GO:1901576) cellular nitrogen compound metabolic process (GO:0034641) regulation of macromolecule metabolic process (GO:0060255) granulocyte activation (GO:0036230) regulation of cellular metabolic process (GO:0031323) positive regulation of response to stimulus (GO:0048584) negative regulation of macromolecule metabolic process (GO:0010605) positive regulation of biological process (GO:0048518) positive regulation of nitrogen compound metabolic process (GO:0051173) myeloid cell activation involved in immune response (GO:0002275) organelle organization (GO:0006996) metabolic process (GO:0008152) regulation of nitrogen compound metabolic process (GO:0051171) neutrophil activation involved in immune response (GO:0002283) negative regulation of cellular process (GO:0048523) negative regulation of biological process (GO:0048519) regulation of protein metabolic process (GO:0051246) negative regulation of metabolic process (GO:0009892) regulation of response to stimulus (GO:0048583) macromolecule biosynthetic process (GO:0009059) localization (GO:0051179) regulation of transcription, DNA-templated (GO:0006355) regulation of cellular protein localization (GO:1903827) phosphorus metabolic process (GO:0006793) nucleobase-containing compound metabolic process (GO:0006139) sensory perception of chemical stimulus (GO:0007606) regulation of organelle organization (GO:0033043) protein-containing complex subunit organization (GO:0043933) response to stress (GO:0006950) hematopoietic or lymphoid organ development (GO:0048534) regulation of catalytic activity (GO:0050790) regulation of response to stress (GO:0080134) detection of stimulus involved in sensory perception (GO:0050906) regulation of protein localization (GO:0032880) regulation of response to external stimulus (GO:0032101) cellular component biogenesis (GO:0044085) sensory perception of smell (GO:0007608) cellular catabolic process (GO:0044248) cellular component assembly (GO:0022607) regulation of intracellular signal transduction (GO:1902531) cellular response to chemical stimulus (GO:0070887) negative regulation of nitrogen compound metabolic process (GO:0051172) regulation of defense response (GO:0031347) positive regulation of macromolecule biosynthetic process (GO:0010557) macromolecule catabolic process (GO:0009057) cellular component organization (GO:0016043) regulation of cellular biosynthetic process (GO:0031326) myeloid leukocyte activation (GO:0002274) hemopoiesis (GO:0030097) organic substance catabolic process (GO:1901575) nucleobase-containing compound biosynthetic process (GO:0034654) immune system development (GO:0002520) positive regulation of cell communication (GO:0010647) positive regulation of programmed cell death (GO:0043068) phagocytosis, recognition (GO:0006910) positive regulation of metabolic process (GO:0009893) regulation of cellular component organization (GO:0051128) detection of stimulus (GO:0051606) regulation of metabolic process (GO:0019222) leukocyte activation (GO:0045321) heterocycle biosynthetic process (GO:0018130) regulation of macromolecule biosynthetic process (GO:0010556) regulation of biological process (GO:0050789) regulation of gene expression (GO:0010468) negative regulation of cellular protein metabolic process (GO:0032269) regulation of nucleic acid-templated transcription (GO:1903506) cell activation (GO:0001775) regulation of nucleobase-containing compound metabolic process (GO:0019219) response to cytokine (GO:0034097) negative regulation of cell death (GO:0060548) negative regulation of cellular metabolic process (GO:0031324) regulation of localization (GO:0032879) regulation of RNA metabolic process (GO:0051252) regulation of biosynthetic process (GO:0009889) protein deubiquitination (GO:0016579) cellular metabolic process (GO:0044237) positive regulation of response to biotic stimulus (GO:0002833) establishment of localization (GO:0051234) heterocycle metabolic process (GO:0046483) sensory perception (GO:0007600) regulation of innate immune response (GO:0045088) regulation of immune system process (GO:0002682) regulation of primary metabolic process (GO:0080090) organic substance metabolic process (GO:0071704) regulation of response to biotic stimulus (GO:0002831) regulation of molecular function (GO:0065009) |
| ADAMTSL4 NOR | 19 | regulation of autophagosome assembly (GO:2000785) antigen processing and presentation of peptide antigen (GO:0048002) regulation of vacuole organization (GO:0044088) antigen processing and presentation (GO:0019882) negative regulation of immune system process (GO:0002683) response to biotic stimulus (GO:0009607) antigen processing and presentation of exogenous antigen (GO:0019884) negative regulation of response to biotic stimulus (GO:0002832) response to chemical (GO:0042221) response to external biotic stimulus (GO:0043207) response to other organism (GO:0051707) cell division (GO:0051301) antigen processing and presentation of exogenous peptide antigen (GO:0002478) regulation of tumor necrosis factor superfamily cytokine production (GO:1903555) negative regulation of macroautophagy (GO:0016242) regulation of tumor necrosis factor production (GO:0032680) defense response (GO:0006952) regulation of peptide transport (GO:0090087) regulation of macroautophagy (GO:0016241) |
| ADAMTSL4 FRA | 315 | complement activation (GO:0006956) small GTPase mediated signal transduction (GO:0007264) regulation of cell adhesion (GO:0030155) mRNA splicing, via spliceosome (GO:0000398) regulation of intracellular protein transport (GO:0033157) homeostasis of number of cells (GO:0048872) nuclear transport (GO:0051169) chromatin organization (GO:0006325) protein polyubiquitination (GO:0000209) negative regulation of biosynthetic process (GO:0009890) negative regulation of transcription by RNA polymerase II (GO:0000122) mitotic cell cycle process (GO:1903047) cellular protein metabolic process (GO:0044267) regulation of Ras protein signal transduction (GO:0046578) G protein-coupled receptor signaling pathway (GO:0007186) negative regulation of cellular catabolic process (GO:0031330) phosphorylation (GO:0016310) nucleobase-containing compound catabolic process (GO:0034655) regulation of autophagy (GO:0010506) rRNA metabolic process (GO:0016072) negative regulation of translation (GO:0017148) regulation of cell cycle (GO:0051726) amide biosynthetic process (GO:0043604) regulation of anatomical structure morphogenesis (GO:0022603) regulation of multicellular organismal development (GO:2000026) cellular response to DNA damage stimulus (GO:0006974) ubiquitin-dependent protein catabolic process (GO:0006511) viral gene expression (GO:0019080) negative regulation of RNA metabolic process (GO:0051253) B cell receptor signaling pathway (GO:0050853) cellular protein localization (GO:0034613) positive regulation of catabolic process (GO:0009896) response to nitrogen compound (GO:1901698) negative regulation of signal transduction (GO:0009968) cell cycle process (GO:0022402) regulation of cell population proliferation (GO:0042127) regulation of viral process (GO:0050792) regulation of hemopoiesis (GO:1903706) nervous system process (GO:0050877) negative regulation of RNA biosynthetic process (GO:1902679) protein localization to endoplasmic reticulum (GO:0070972) histone modification (GO:0016570) nuclear export (GO:0051168) regulation of type I interferon production (GO:0032479) RNA splicing (GO:0008380) multicellular organism development (GO:0007275) protein-containing complex localization (GO:0031503) nuclear-transcribed mRNA catabolic process, nonsense-mediated decay (GO:0000184) negative regulation of transcription, DNA-templated (GO:0045892) negative regulation of phosphate metabolic process (GO:0045936) activation of innate immune response (GO:0002218) autophagy (GO:0006914) protein localization to organelle (GO:0033365) negative regulation of cell cycle process (GO:0010948) negative regulation of phosphorus metabolic process (GO:0010563) translation (GO:0006412) nucleobase-containing compound transport (GO:0015931) carbohydrate derivative metabolic process (GO:1901135) exocytosis (GO:0006887) translational initiation (GO:0006413) T cell activation (GO:0042110) protein modification by small protein conjugation (GO:0032446) process utilizing autophagic mechanism (GO:0061919) RNA localization (GO:0006403) regulation of cell cycle process (GO:0010564) protein phosphorylation (GO:0006468) cellular nitrogen compound catabolic process (GO:0044270) membrane invagination (GO:0010324) regulation of hydrolase activity (GO:0051336) negative regulation of protein modification process (GO:0031400) mRNA metabolic process (GO:0016071) nucleic acid-templated transcription (GO:0097659) interspecies interaction between organisms (GO:0044419) positive regulation of transcription, DNA-templated (GO:0045893) positive regulation of nucleic acid-templated transcription (GO:1903508) innate immune response-activating signal transduction (GO:0002758) regulation of RNA stability (GO:0043487) negative regulation of programmed cell death (GO:0043069) proteolysis involved in cellular protein catabolic process (GO:0051603) protein targeting to membrane (GO:0006612) positive regulation of intracellular transport (GO:0032388) multi-organism process (GO:0051704) positive regulation of transport (GO:0051050) intracellular protein transport (GO:0006886) RNA metabolic process (GO:0016070) negative regulation of apoptotic process (GO:0043066) response to endogenous stimulus (GO:0009719) cellular response to endogenous stimulus (GO:0071495) regulation of protein phosphorylation (GO:0001932) keratinocyte differentiation (GO:0030216) regulation of vesicle-mediated transport (GO:0060627) macromolecule modification (GO:0043412) regulation of complement activation (GO:0030449) regulation of multicellular organismal process (GO:0051239) lymphocyte mediated immunity (GO:0002449) defense response to bacterium (GO:0042742) regulation of protein kinase activity (GO:0045859) phagocytosis, engulfment (GO:0006911) organonitrogen compound catabolic process (GO:1901565) establishment of organelle localization (GO:0051656) posttranscriptional regulation of gene expression (GO:0010608) protein localization to membrane (GO:0072657) anatomical structure development (GO:0048856) organonitrogen compound biosynthetic process (GO:1901566) regulation of mRNA metabolic process (GO:1903311) gene expression (GO:0010467) peptide transport (GO:0015833) positive regulation of cellular biosynthetic process (GO:0031328) humoral immune response mediated by circulating immunoglobulin (GO:0002455) ribonucleoprotein complex biogenesis (GO:0022613) regulation of apoptotic signaling pathway (GO:2001233) epidermal cell differentiation (GO:0009913) negative regulation of cellular macromolecule biosynthetic process (GO:2000113) aromatic compound catabolic process (GO:0019439) actin filament-based process (GO:0030029) plasma membrane invagination (GO:0099024) system development (GO:0048731) negative regulation of signaling (GO:0023057) protein metabolic process (GO:0019538) peptidyl-lysine modification (GO:0018205) organophosphate biosynthetic process (GO:0090407) DNA repair (GO:0006281) regulation of mitotic cell cycle (GO:0007346) negative regulation of protein phosphorylation (GO:0001933) regulation of binding (GO:0051098) cellular component disassembly (GO:0022411) positive regulation of protein phosphorylation (GO:0001934) establishment of protein localization to organelle (GO:0072594) modification-dependent macromolecule catabolic process (GO:0043632) regulation of developmental process (GO:0050793) positive regulation of proteolysis (GO:0045862) regulation of mRNA processing (GO:0050684) positive regulation of gene expression (GO:0010628) organelle localization (GO:0051640) establishment of protein localization (GO:0045184) cellular response to stimulus (GO:0051716) regulated exocytosis (GO:0045055) cellular response to cytokine stimulus (GO:0071345) positive regulation of protein modification process (GO:0031401) regulation of intracellular transport (GO:0032386) cell activation involved in immune response (GO:0002263) RNA transport (GO:0050658) positive regulation of protein metabolic process (GO:0051247) negative regulation of gene expression (GO:0010629) negative regulation of cell communication (GO:0010648) RNA splicing, via transesterification reactions (GO:0000375) positive regulation of multi-organism process (GO:0043902) lymphocyte differentiation (GO:0030098) immunoglobulin mediated immune response (GO:0016064) negative regulation of response to stimulus (GO:0048585) transcription by RNA polymerase II (GO:0006366) peptidyl-amino acid modification (GO:0018193) proteasomal protein catabolic process (GO:0010498) positive regulation of phosphorus metabolic process (GO:0010562) establishment of RNA localization (GO:0051236) positive regulation of developmental process (GO:0051094) negative regulation of cellular biosynthetic process (GO:0031327) negative regulation of mitotic cell cycle (GO:0045930) RNA splicing, via transesterification reactions with bulged adenosine as nucleophile (GO:0000377) regulation of phosphorylation (GO:0042325) actin cytoskeleton organization (GO:0030036) humoral immune response (GO:0006959) proteolysis (GO:0006508) rRNA processing (GO:0006364) peptide metabolic process (GO:0006518) viral transcription (GO:0019083) chromosome organization (GO:0051276) regulation of cellular localization (GO:0060341) epidermis development (GO:0008544) response to abiotic stimulus (GO:0009628) regulation of symbiosis, encompassing mutualism through parasitism (GO:0043903) positive regulation of RNA biosynthetic process (GO:1902680) regulation of protein catabolic process (GO:0042176) response to endoplasmic reticulum stress (GO:0034976) regulation of protein serine/threonine kinase activity (GO:0071900) programmed cell death (GO:0012501) SRP-dependent cotranslational protein targeting to membrane (GO:0006614) protein targeting to ER (GO:0045047) apoptotic process (GO:0006915) secretion (GO:0046903) cytokine-mediated signaling pathway (GO:0019221) regulation of mitotic cell cycle phase transition (GO:1901990) positive regulation of cellular protein metabolic process (GO:0032270) protein export from nucleus (GO:0006611) negative regulation of macromolecule biosynthetic process (GO:0010558) mitotic cell cycle (GO:0000278) cellular response to external stimulus (GO:0071496) peptide biosynthetic process (GO:0043043) regulation of protein stability (GO:0031647) cellular amide metabolic process (GO:0043603) regulation of cellular amide metabolic process (GO:0034248) ncRNA processing (GO:0034470) RNA processing (GO:0006396) protein-containing complex disassembly (GO:0032984) ribosome biogenesis (GO:0042254) negative regulation of catabolic process (GO:0009895) mRNA processing (GO:0006397) regulation of cellular component biogenesis (GO:0044087) negative regulation of cell cycle (GO:0045786) B cell mediated immunity (GO:0019724) regulation of mRNA catabolic process (GO:0061013) post-Golgi vesicle-mediated transport (GO:0006892) small molecule metabolic process (GO:0044281) negative regulation of nucleic acid-templated transcription (GO:1903507) positive regulation of phosphorylation (GO:0042327) negative regulation of organelle organization (GO:0010639) ribonucleoprotein complex subunit organization (GO:0071826) RNA biosynthetic process (GO:0032774) regulation of humoral immune response (GO:0002920) ncRNA metabolic process (GO:0034660) cotranslational protein targeting to membrane (GO:0006613) regulation of protein modification process (GO:0031399) regulation of cell differentiation (GO:0045595) regulation of translation (GO:0006417) regulation of cell cycle phase transition (GO:1901987) cell death (GO:0008219) regulation of chromosome organization (GO:0033044) cell surface receptor signaling pathway (GO:0007166) regulation of phosphate metabolic process (GO:0019220) modification-dependent protein catabolic process (GO:0019941) response to stimulus (GO:0050896) cellular protein-containing complex assembly (GO:0034622) ribonucleoprotein complex assembly (GO:0022618) regulation of transferase activity (GO:0051338) protein catabolic process (GO:0030163) regulation of transport (GO:0051049) leukocyte activation involved in immune response (GO:0002366) viral process (GO:0016032) endomembrane system organization (GO:0010256) positive regulation of protein kinase activity (GO:0045860) positive regulation of innate immune response (GO:0045089) regulation of cytoskeleton organization (GO:0051493) positive regulation of hydrolase activity (GO:0051345) positive regulation of kinase activity (GO:0033674) response to organonitrogen compound (GO:0010243) positive regulation of cellular component organization (GO:0051130) RNA export from nucleus (GO:0006405) protein modification by small protein conjugation or removal (GO:0070647) membrane organization (GO:0061024) cellular response to organic cyclic compound (GO:0071407) cornification (GO:0070268) developmental process (GO:0032502) response to oxygen-containing compound (GO:1901700) positive regulation of molecular function (GO:0044093) system process (GO:0003008) positive regulation of cellular catabolic process (GO:0031331) regulation of protein complex assembly (GO:0043254) heterocycle catabolic process (GO:0046700) cytoskeleton organization (GO:0007010) nucleic acid metabolic process (GO:0090304) establishment of protein localization to membrane (GO:0090150) DNA metabolic process (GO:0006259) proteasome-mediated ubiquitin-dependent protein catabolic process (GO:0043161) transcription, DNA-templated (GO:0006351) regulation of mitochondrion organization (GO:0010821) regulation of mRNA stability (GO:0043488) nuclear-transcribed mRNA catabolic process (GO:0000956) Golgi vesicle transport (GO:0048193) regulation of cellular response to stress (GO:0080135) regulation of phosphorus metabolic process (GO:0051174) nucleic acid transport (GO:0050657) nucleocytoplasmic transport (GO:0006913) regulation of GTPase activity (GO:0043087) regulation of response to DNA damage stimulus (GO:2001020) amide transport (GO:0042886) establishment of protein localization to endoplasmic reticulum (GO:0072599) symbiotic process (GO:0044403) positive regulation of transcription by RNA polymerase II (GO:0045944) positive regulation of catalytic activity (GO:0043085) regulation of kinase activity (GO:0043549) nervous system development (GO:0007399) protein modification process (GO:0036211) negative regulation of intracellular signal transduction (GO:1902532) positive regulation of defense response (GO:0031349) leukocyte differentiation (GO:0002521) negative regulation of cellular component organization (GO:0051129) export from cell (GO:0140352) intracellular transport (GO:0046907) cellular protein catabolic process (GO:0044257) regulation of small GTPase mediated signal transduction (GO:0051056) organic cyclic compound catabolic process (GO:1901361) homeostatic process (GO:0042592) positive regulation of RNA metabolic process (GO:0051254) regulation of DNA metabolic process (GO:0051052) RNA catabolic process (GO:0006401) skin development (GO:0043588) cellular protein modification process (GO:0006464) regulation of RNA splicing (GO:0043484) cellular macromolecule localization (GO:0070727) regulation of gene expression, epigenetic (GO:0040029) myeloid leukocyte mediated immunity (GO:0002444) covalent chromatin modification (GO:0016569) protein targeting (GO:0006605) adaptive immune response (GO:0002250) lymphocyte activation (GO:0046649) positive regulation of biosynthetic process (GO:0009891) negative regulation of phosphorylation (GO:0042326) regulation of lipid metabolic process (GO:0019216) positive regulation of multicellular organismal process (GO:0051240) negative regulation of nucleobase-containing compound metabolic process (GO:0045934) negative regulation of cellular amide metabolic process (GO:0034249) positive regulation of transferase activity (GO:0051347) protein transport (GO:0015031) organophosphate metabolic process (GO:0019637) endosomal transport (GO:0016197) protein ubiquitination (GO:0016567) DNA replication (GO:0006260) positive regulation of organelle organization (GO:0010638) secretion by cell (GO:0032940) complement activation, classical pathway (GO:0006958) positive regulation of cellular protein localization (GO:1903829) regulation of transcription by RNA polymerase II (GO:0006357) positive regulation of phosphate metabolic process (GO:0045937) mRNA catabolic process (GO:0006402) positive regulation of nucleobase-containing compound metabolic process (GO:0045935) |
